# Safe guidewire visualization using the modes of a PTx transmit array MR system

**DOI:** 10.1002/mrm.28069

**Published:** 2019-11-13

**Authors:** Felipe Godinez, Greig Scott, Francesco Padormo, Joseph V. Hajnal, Shaihan J. Malik

**Affiliations:** ^1^ School of Biomedical Engineering and Imaging Sciences King’s College London London United Kingdom; ^2^ Magnetic Resonance Systems Research Laboratory Department of Electrical Engineering Stanford University Stanford California USA; ^3^ Guy’s and St. Thomas’ NHS Foundation Trust London United Kingdom

**Keywords:** auxiliary PTx system, cardiac catheters, guidewire visualization, interventional MRI catheterization, invasive hemodynamics, medical device heating, parallel transmit MRI, real‐time MRI

## Abstract

**Purpose:**

MRI‐guided cardiovascular intervention using standard metal guidewires can produce focal tissue heating caused by induced radiofrequency guidewire currents. It has been shown that safe operation is made possible by using parallel transmit radiofrequency coils driven in the null current mode, which does not induce radiofrequency currents and hence allows safe tissue visualization. We propose that the maximum current modes, usually considered unsafe, be used at very low power levels to visualize conductive wires, and we investigate pulse sequences best suited for this application.

**Methods:**

Spoiled gradient echo, balanced steady‐state free precession, and turbo spin echo sequences were evaluated for their ability to visualize a conductive guidewire embedded in a gel phantom when run in maximum current modes at very low power level. Temperature at the guidewire tip was monitored for safety assessment.

**Results:**

Excellent guidewire visualization could be achieved using maximum current modes excitation, with the turbo spin echo sequence giving the best image quality. Although turbo spin echo is usually considered to be a high‐power sequence, our method reduced all pulses to 1% amplitude (0.01% power), and heating was not detected. In addition, visualization of background tissue can be achieved using null current mode, also with no recorded heating at the guidewire tip even when running at 100% (reported) specific absorption rate.

**Conclusion:**

Parallel transmit is a promising approach for both guidewire and tissue visualization using maximum and null current modes, respectively, for interventional cardiac MRI. Such systems can switch excitation mode instantaneously, allowing for flexible integration into interactive sequences.

## 1 INTRODUCTION

Catheter‐based procedures in cardiovascular interventions are typically guided under X‐ray fluoroscopy to visualize the guidewire‐catheter system within the surrounding anatomical structures. The use of MRI for guidance leads to both improved soft tissue contrast and elimination of radiation dose.^1^, ^2^ However, many existing interventional devices used under X‐ray guidance are made of metal or contain metal and thus are electrically conductive and may be ferrous. Ferrous devices are contraindicated for use in MRI systems either because of excessive forces or production of unacceptable levels of artifact. However, even nonferrous metallic devices are susceptible to radiofrequency (RF) induced currents, which can lead to dangerous focal heating of tissues. This is a well‐known risk for MRI.^3^, ^4^, ^5^ It has been shown that the key variables associated with guidewire safety in MRI are the guidewire diameter, total length, insertion length, and volume of the dielectric medium.^6^, ^7^ In the clinical scenario during an intervention, the insertion length can be constantly changing, which emphasizes the need to control the RF‐induced current on the guidewire dynamically.

Many approaches have been explored to overcome the problem of heating of interventional devices. The simplest strategy is to use unmodified nonferrous devices with conventional scanners operating with lower RF transmission levels. Although effective, this often results in compromised imaging efficiency and unfavorable tradeoffs in visualization of both anatomy and devices.^8^ An alternative approach is to modify the devices themselves so they cannot support RF currents. However, this approach requires a new generation of interventional devices that do not jeopardize mechanical performance.^9^

In addition to guidewire safety, the inability to robustly visualize standard guidewires presently prevents the ubiquitous practice of MRI‐guided interventions. Therefore, guidewire visualization has been a focus of current research. State‐of‐the‐art guidewire visualization methods primarily rely on either susceptibility‐related effects,^10^, ^11^ auxiliary contrast markers,^12^, ^13^ or some form of local signal detector mounted on or integrated into the device.^14^, ^15^, ^16^ Moreover, pulse sequences and RF field polarization methods exist that allow device‐tracking without the need of modifications.^17^, ^18^ For instance, Campbell‐Washburn et al^10^ harnessed susceptibility effects using a gradient echo sequence in conjunction with through‐slice dephasing to make the guidewire appear hyperintense while suppressing background signal. However, sharp changes in susceptibility also exist at air and tissue interfaces, which can also appear hyperintense, thus interfering with the segmentation of the guidewire from the background. To overcome these limitations, hardware solutions capable of detecting the NMR signal adjacent to the device have been introduced. These solutions utilize active components, such as saddle coils, loopless antennae, and solenoid coils acting as receive coils^9^, ^13^, ^19^; alternately, they utilize passive components, such as chokes and resonant networks, to change the RF properties of the device at the Larmor frequency.^20^ In 1 example, Etezadi‐Amoli et al^14^ placed a toroidal coil over a guidewire to excite and receive local NMR signals. Solutions for the safety and visualization of catheters have also been attempted on guidewires.^10^, ^21^, ^22^, ^23^ For instance, Sonmez et al^23^ attached an active solenoid coil for visualization and a temperature probe for temperature monitoring, modifying the guidewire substantially.

Additional opportunities toward safe device visualization may be found in harnessing and controlling the typicaly dangerous RF‐induced currents. These behave according to Ampère’s law and enhance the RF magnetic field (*B*_1_) around the guidewire mediating the visualization. The control of the RF‐induced current may be achieved by manipulating the electromagnetic fields around the guidewire, given that the currents depend on the magnitude and phase of the incident electric field. A degree of control over the emitted electromagnetic fields is possible with parallel transmit (PTx) array coils. It has been shown that RF‐induced current “modes” may exist on the guidewire and can be determined by 1 or more current sensors placed over the guidewire.^24^ Typically, 1 or more maximum current modes (MM) in which the PTx system produces a strong current on the conductor exist, along with additional null current modes (NM)^24^, ^25^, ^26^ in which RF excitation produces 0 measured current at the sensor’s location. Despite the NMs producing little or no RF current, they can still generate substantial transmit ($B_{1}^{+}$) field; hence, they can be harnessed for safe imaging of the anatomical structures with a guidewire in situ. In addition to the cardiac interventional example that is the focus of this work, NMs have been explored for other safe imaging applications, including the imaging of deep brain stimulators.^25^, ^26^, ^27^ On the other hand, the MMs can produce dangerously high RF currents that enhance the transmit $B_{1}^{+}$ fields near the guidewire. MMs have not been used for imaging because of their potential for heating; but if properly treated, these modes can help in visualizing the guidewire by directly imaging the NMR adjacent to the guidewire. The magnitude of the $B_{1}^{+}$ produced is inversely proportional to the radial distance from the guidewire; thus, even small guidewire currents can produce significant $B_{1}^{+}$ adjacent to it. With very low RF power sequences, a measurable signal comes mainly from the locality of the guidewire.^27^, ^28^ Some have shown that the guidewire to coil coupling can be leveraged for visualization using birdcage coils and that it can be used to assess the safety of RF excitation.^27^, ^29^ The combination of PTx control and direct RF current monitoring of induced currents may provide an alternative means to both visualize the device and anatomy in a safe manner without device modification.

The work by Etezadi‐Amoli et al^24^ has shown that coupling modes exist and has characterized them. The main focus of this paper is to demonstrate that the coupling mode MM (usually omitted and considered hazardous) can be harnessed for safe and robust guidewire visualization, as well as to investigate the performance of common pulse sequence types for optimum device visualization in this regime.

## 2 THEORY

### 2.1 Transmit field enhancement

**Figure 1:**
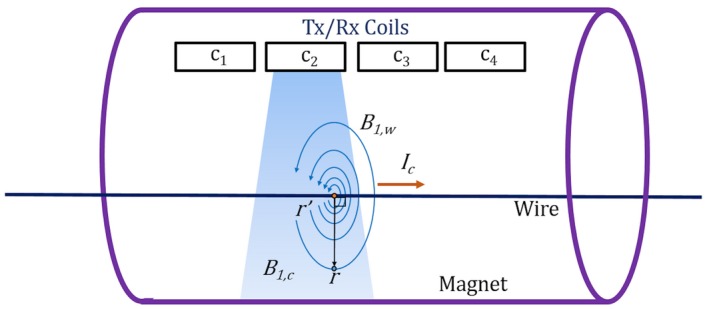
Schematic illustrating the *B*_1_ field enhancement near the guidewire

The electromagnetic RF field used during MRI transmission consists of both a magnetic field, $B_{1}^{+}$, and an electric field, *E*. The electric field drives currents, *I*, on the guidewire, which in turn generate a local magnetic field, *B*_1,_ *_w_*; they also produce heating at locations where charge can be displaced in the dielectric medium, for example at the guidewire tip.^20^ The transmitted magnetic field is enhanced or suppressed by the local magnetic field, thus generating NMR signal predominantly from protons around the guidewire. This enhancement can be large enough so that with low RF power a detectable NMR signal is produced using standard pulse imaging sequences, which enables safe guidewire visualization. In a PTx system, whereas the transmit magnetic field $B_{1,c}^{+}\left(r\right)$, at location r, is produced by the c^th^ element, the simultaneously produced electric field *E_c_*(*r*) will induce current *I_c_*(*r*′), at location *r*′, on a conductive guidewire (Figure 1). This induced current in turn generates a local scattered magnetic field, *B*_1,_ *_w_*(*r*), which is linearly polarized and oriented in the circumferential direction.^30^, ^31^ This field adds together with the imposed $B_{1,c}^{+}$ field produced by the RF coil. We define the guidewire enhancement factor *k*(*r*) as the ratio of the $B_{1}^{+}$ field produced with the guidewire in, compared with the guidewire out, such that sufficiently close to the guidewire, k≫1.

When transmitting on all channels simultaneously, the overall $B_{1}^{+}$ field is a linear combination of the individual coil $B_{1,c}^{+}$ fields weighted by complex weighting factors *w_c_*, often referred to as RF shims. This is also true for the induced current on the guidewire because it is linearly related to the fields produced by each coil. The MM mode (the mode with the largest singular value) has the property of maximizing the induced current *I* and thus will also maximize *k*(*r*). The method of mode identification is described in^24^: briefly, modes are computed by the singular value decomposition of the coupling matrix, which is formed from current measurements obtained by different current sensors when the RF array is driven 1 coil at a time with the same amplitude and phase. In the MM mode, the flip angle produced by a given RF pulse is now also a strong spatially dependent function as a result of the spatially variable fields. To avoid confusion, we will define the nominal flip angle *θ* as the angle that would be achieved in the absence of any wires. If the local $B_{1}^{+}$ field has been significantly enhanced, the true flip angle will be much greater. Hence, even when using low amplitude pulses that do not create significant risk of heating, high flip angles can be produced close to a conductor using the MM mode, enabling visualization of the guidewire while generating limited signal from the rest of the object. Optimum guidewire visualization may contain the following properties: no heating risk, low signal in the background at the nominal flip angle, large signal enhancement at the guidewire (a large enhancement factor k), and short time per image frame to allow dynamic imaging of moving guidewires.

### 2.2 Sequences for guidewire visualization

The following pulse sequences were considered and chosen for their rapid imaging ability: balanced steady state free precession (bSSFP), spoiled gradient echo (SPGR), single shot turbo spin echo (TSE), and single shot echo planar imaging (EPI). The signal response for each of these can be compared by numerical simulation, assuming a reference T_1_ time of 1531 ms^32^ and T_2_ of 100 ms, approximately relevant to blood at 1.5 Tesla.^33^ For the sake of comparison, we consider 2D versions of all sequences and a scenario where the repetition time (TR) for bSSFP and SPGR and the echo spacing for the TSE sequence are all the same; this was fixed to 4 ms. Whereas the short TR sequences can be studied in steady state, this is not so for TSE; thus, we adopted a plausible single shot imaging scenario assuming an echo train length of 50 echoes, with partial Fourier encoding such that the signal is determined by the 10th echo. Because dynamic repetition of TSE leads to saturation, we include a 100 ms delay between shots and a tip‐back pulse to speed up longitudinal recovery.^34^ For TSE, we define *θ* as the excitation nominal flip angle and use refocusing flip angle 2*θ* with a flip‐back pulse at the end of the echo train of angle −*θ*. The EPI sequence does not have a simple comparison point in terms of numbers of RF pulses; hence, it was simulated with TR 200 ms, which corresponds to a similar frame rate to the other methods. Simulated signals were computed using known steady‐state expressions for the SPGR/EPI (i.e., the Ernst equation) and for bSSFP. TSE was simulated using the extended phase graph^35^ method, with the steady state over multiple dynamics computed as described in Ref. 36.

**Figure 2:**
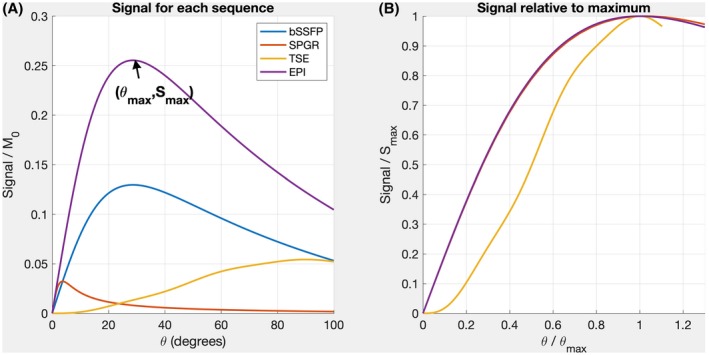
(A) Predicted signals relative to *M*_0_ for each candidate sequence (details in text); for TSE, the excitation flip angle is *θ*, and the refocusing angle is 2*θ*. For each signal trace, the peak signal *S_max_* occurs at flip angle *θ_max_*. (B) Predicted signals normalized to *S_max_* versus flip angle normalized to *θ_max_*. This plot shows how the signal drops off as the flip angle is reduced from *θ_max_* for each sequence. TSE, turbo spin echo

Figure 2 contains the results of these simulations. Figure 2A shows the signal as a function of flip angle. Each curve has a maximum signal, *S_max_*, occurring at some flip angle *θ_max_*. If these sequences are used with a MM RF shim setting, the flip angle local to the guidewire will be much larger than the surroundings. If we adjust the power level such that the flip angle local to the guidewire is *θ_max_*, this will maximize the signal from the guidewire. With these considerations in mind, we can conclude that the best sequence in terms of overall signal to noise ratio (SNR) is likely to be EPI, with bSSFP coming second. We may, however, exclude EPI from the candidates because in reality the high degree of *B*_0_ field inhomogeneity that can be expected over wide field of view (FOV) required for this application would lead to spatial distortion and signal loss in this sequence. Figure 2B plots signal normalized to the maximum for each sequence against flip angle normalized by *θ_max_*; this allows us to deduce how quickly the signal will drop off away from the guidewire. A rapid drop‐off in signal will give the sharpest depiction of the guidewire. Figure 2B suggests that the optimal sequence for sharpness in guidewire visualization is the TSE, whereas bSSFP is the optimal sequence to maximize SNR.

## 3 METHODS

### 3.1 Hardware and phantoms

Experiments were performed by measuring currents on a standard guidewire in a gel phantom in the presence of RF excitation. All measurements were performed on a 3 Tesla MRI system (Achieva, Philips, Netherlands) with an 8‐channel transceiver transverse electromagnetic body coil^37^ used for transmission and a local 6‐channel torso coil for reception. A 14‐liter phantom was filled with poly(acrylic) acid gel prepared according to American Society for Testing and Materials standard F2182.^38^ An array of horizontally tilted plastic circles and an Eppendorf tube (filled with mineral oil, the narrow end colocalized with the guidewire tip) were placed in the gel to add distinguishable features. The relaxation properties of the gel were measured using a 1 liter sample studied with inversion recovery TSE for T_1_ and multi‐echo spin echo for T_2_. The measured parameters were T_1_ = 2270 ms, T_2_ = 250 ms.

Experiments used a standard nitinol core guidewire with polyurethane outer coating of 0.89 mm diameter and cut to 900 mm length (RF+GA35153M, Terumo Corporation, Japan) to increase resonance. The most distal 4 mm of the guidewire’s polyurethane coating was removed because an exposed tip is expected to produce a worst‐case heating condition^4^ and was not done so for visualization gains. The guidewire was placed in the phantom oriented along the static magnetic field direction of the scanner, with 46 cm of the guidewire immersed in the gel. RF current measurements were made using 2 toroidal coil sensors,^14^ both placed over the guidewire outside the gel. The sensors used an electro‐optical connection to minimize direct coupling to the RF fields^14^; RF signals were measured directly by the scanner’s spectrometer.

### 3.2 Current modes and RF shimming

As outlined above, the maximum and null current modes were determined, as described by Etezadi‐Amoli et al,^24^ using the measured induced RF current on the guidewire. A 2 × 8 coupling matrix was formed by measuring induced currents from the 2 sensors as each of the 8 transmit channels was energized in turn. After performing the singular value decomposition on the coupling matrix, the columns of the right singular vector’s matrix yielded the required mode weights with 2 MMs and 6 NMs. This is because the coupling matrix has a rank of 2, giving 2 singular vectors with non‐0 singular values and 6 with 0 singular value forming the null space.^24^ The mode with the largest singular value was chosen as the MM RF shim and used to visualize the guidewire only. For tissue visualization, a uniform $B_{1}^{+}$ field must be constructed from the remaining null modes because each produces 0 induced currents; any linear combination will produce the same result. By using the actual flip‐angle method^39^ in a combined (nominal quadrature) mode and using the low flip angle SPGR scans for each individual channel, $B_{1}^{+}$ maps for each coil were acquired. The resulting per channel $B_{1}^{+}$ maps form the columns of N × 8 matrix *P*, where N is the number of pixels in each image. The 6 × 8 matrix $\tilde{V}$ of channel weights for each of the 6 null modes may then be used to compute $\tilde{P}\;=\;P\tilde{V}$, which is a matrix of null mode $B_{1}^{+}$ maps. These were used within a magnitude‐least‐squares RF shimming calculation^40^:

(1) $$\min_{x}\left\{{∥\left|\tilde{P}x\right|-T∥}^{2}\right\}$$

(2) $$w=\tilde{V}x,$$

where x is a vector of complex RF shim weights to apply to each virtual null mode, whereas w is a vector of complex RF shim weights to apply to each physical coil, and *T* is the target $B_{1}^{+}$. The goal of the optimization was a uniform field with a magnitude equivalent to generate 100% of the nominal flip angle. Note that, although the optimization computes x, the shims w are actually applied to the coil.

### 3.3 Determination of guidewire enhancement factor

The guidewire enhancement factor *k* was measured by acquiring multiple SPGR images with a range of 64 different nominal flip angles *θ* with the guidewire in and out. In each case, the measured signals *S* were fitted pixel‐wise to Equation 3:

(3) $$S=A\frac{\sin\left(c\theta \right)\left(1-e^{-\frac{\mathrm{TR}}{T_{1}}}\right)}{1-\cos\left(c\theta \right)e^{-\frac{\mathrm{TR}}{T_{1}}}},$$

where the local $B_{1}^{+}$ scaling factor *c* and overall scaling *A* were the unknowns. The guidewire enhancement factor is then given by the ratio of *c* with guidewire‐in to guidewire‐out. In these experiments, a 5 mm slice orthogonal to the guidewire was used, with 1 mm in plane resolution and TR = 3.3 ms. For the guidewire‐in, *θ* was stepped in the ranges 0° to 5° in steps of 0.08° and then 5° to 90° in steps of 2.75°, and for guidewire‐out in the ranges 0° to 90° in steps of 2.9°. Uncertainty in the estimated enhancement factors was estimated using a residual resampling bootstrapping method.

### 3.4 Imaging experiments

Visualization of the guidewire was performed using MM excitation with low power, a mode usually not considered safe at normal power levels. The candidate pulse sequences (SPGR, bSSFP, and TSE) were all set up with a thick slice (50 mm) to produce a projection image over the complete guidewire region. Sequences were individually optimized to maximize frame rate for the same nominal resolution (1 mm in plane) and FOV (252 mm × 398 mm). For the SPGR sequence, the echo time (TE) and the TR were 1.54 ms and 3.19 ms, respectively, whereas for the bSSFP these were 1.55 ms and 3.10 ms, respectively. The bSSFP used SENSE factor 2 and partial Fourier reconstruction to obtain a frame rate of 2.5 frames per second (fps); these acceleration measures were not used for the SPGR because the SNR was insufficient, so this yielded a frame rate of 0.79 fps. The single shot TSE used an echo spacing of 4.8 ms and a combination of SENSE factor 2 and partial Fourier reconstruction to give an TE of 53 ms and frame rate of 1.01 fps. Note that flip‐back pulses were not used for the TSE in experiments because they were not found to have a strong influence at the frame rates used here.

Although the PTx system can be used to set the maximum current mode, the guidewire enhancement factor is not known in advance; thus, the optimal nominal flip angle for visualization must be determined empirically for each sequence. This was done by sweeping through a range of input power scales and selecting the best power level empirically based on guidewire visualization. The criteria used for this were 1) sharp depiction of the guidewire shaft, 2) as much of the guidewire tip visible as possible, and 3) minimal background signal.

**Figure 3:**
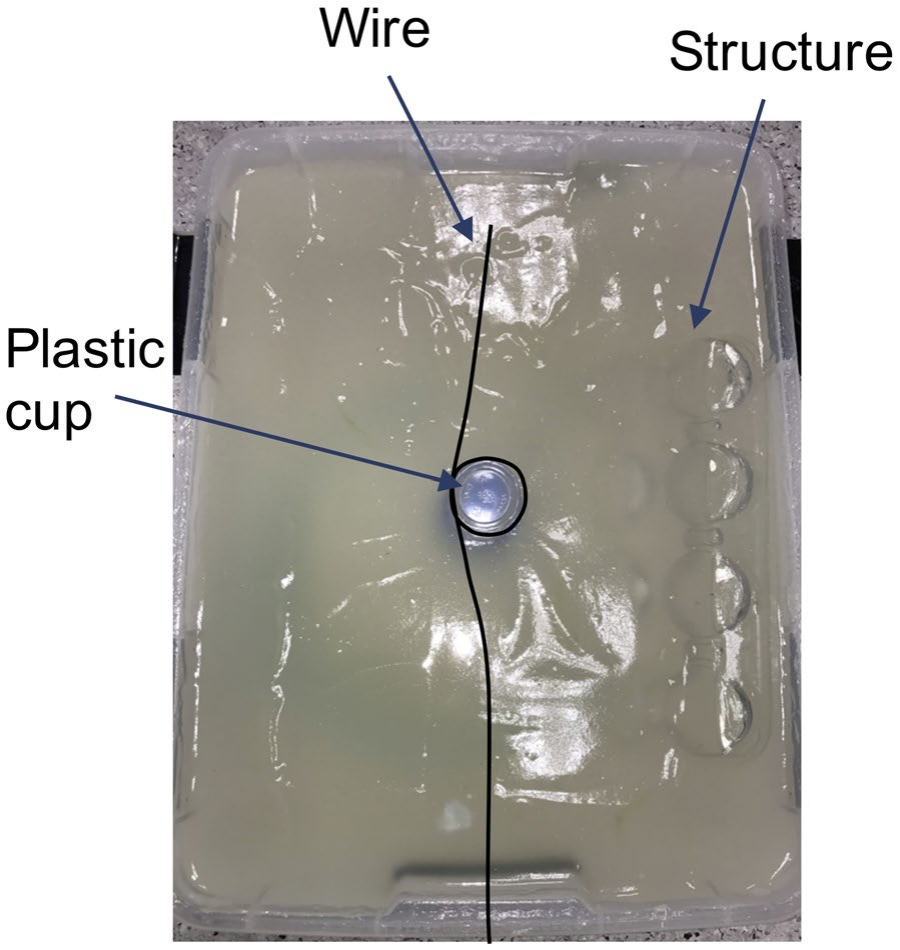
The guidewire geometry used while the guidewire was withdrawn from the phantom

For the real‐time visualization test, a 150 cm long guidewire was pulled out of the phantom during imaging with TSE. An approximately 5 cm diameter loop was formed by the guidewire in the FOV (Figure 3). To speed up the frame rate to 2.5 fps, a lower resolution of 2 mm was used. The guidewire slowly was pulled out manually while the imaging protocol dynamically repeated for 100 frames.

### 3.5 Guidewire heating tests

During imaging protocols, temperature at the bare tip of the guidewire was monitored using a fiber‐optic temperature probe (LumaSense Technologies, Inc., USA) attached to the guidewire tip, tied on with nylon string in parallel with the guidewire axis and flush with the guidewire tip. It is shown in the literature that the guidewire tip is the site for worst case heating^20^; it has recently also been demonstrated from a study in the presence of deep brain stimulator electrodes that RF did not increase the heating or specific absorption rate (SAR) at other locations far from the wire tip.^25^ Worst case heating was demonstrated by running a TSE scan at high power (maximum allowable scanner‐reported SAR for 6 min). Subsequently, equivalent measurements were made while visualization sequences were run. After each heating acquisition, the temperature was allowed to return back to baseline (18.6°C–19.0°C).

## 4 RESULTS

### 4.1 Determination of guidewire enhancement factor

**Figure 4:**
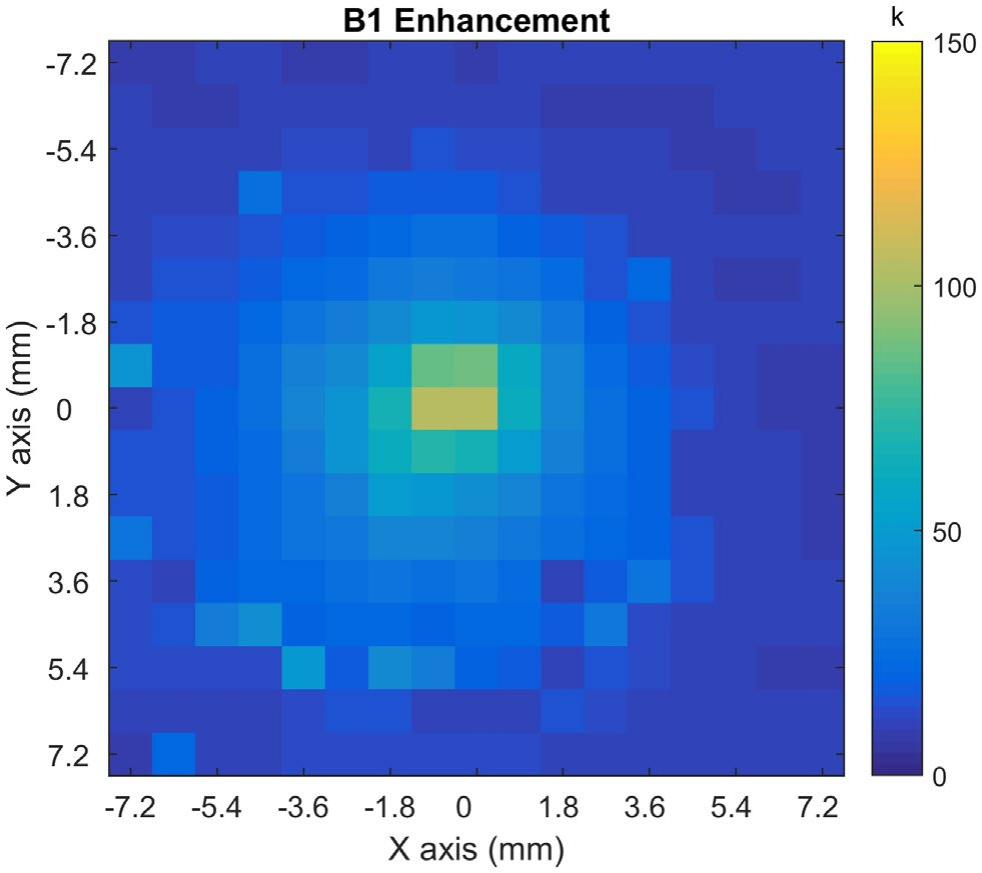
Scale factor, *k*, for *B_1_* enhancement at the guidewire in an axial image (axis labels are in mm). The *B*_1_ enhancement factor is the ratio of the peak signal flip angle with guidewire‐in to guidewire‐out. The pixel size is 0.9 mm × 0.9 mm

A map of the guidewire enhancement factor is shown in Figure 4. The enhancement factor decreases very quickly as distance from the guidewire increases, with the pixel at the guidewire having a maximum enhancement factor of 108 (± 6.6). The mean of 8 central pixels around the guidewire was 82 (± 1.79).

### 4.2 Imaging experiments

**Figure 5:**
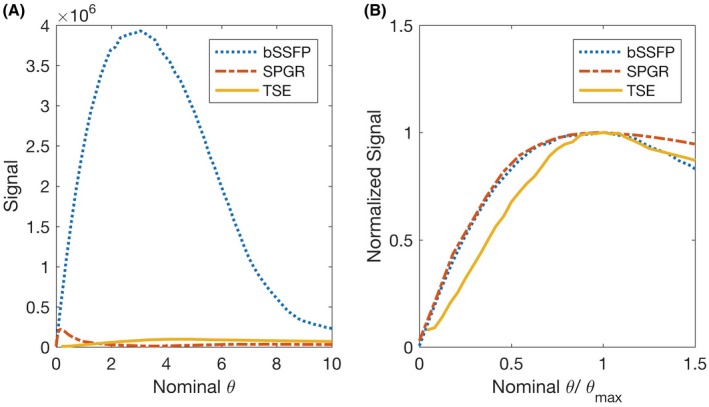
Signal from a pixel at the guidewire location. (A) Measured signals for each candidate sequence using a straight guidewire; for TSE, the excitation flip angle is *θ*, and refocusing angle is 2*θ*. For each signal trace, the peak signal *S_max_* occurs at flip angle *θ_max_*. (B) Measured signals normalized to *S_max_* versus flip angle normalized *θ_max_* using a straight guidewire

The signal as a function of nominal flip angle for each tested sequence is shown in Figure 5, taken from a pixel location close to the guidewire. The shapes of the curves in Figure 5B follow the same profile seen in simulation (Figure 2B), with the SSFP having the highest signal; however, the TSE showing the fastest descent from the peak signal as flip angle is reduced. The nominal flip angle that gives the best guidewire visualization in MM mode was determined to be 0.36°, 0.06°, and 0.94° for bSSFP, SPGR, and TSE, respectively. It should be noted that these values do not correspond to the signal peaks as shown in Figure 5 because the criteria used for determining the nominal flip angle to use for guidewire visualization (described above) considered other factors in addition to SNR.

**Figure 6:**
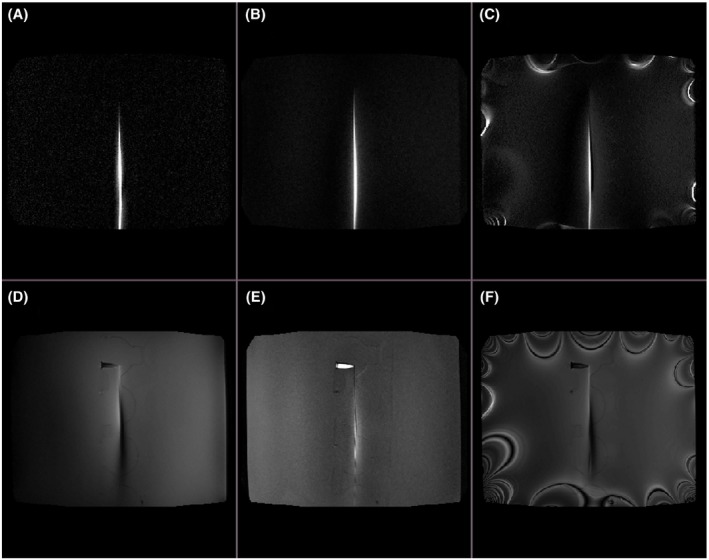
Coronal view of projection images through 50 mm of phantom. The images (A) through (C) were acquired with the MM mode and (D) through (F) with the NM mode optimized for a uniform *B*_1_. (A) TSE (MM) using flip angle 0.94° (excitation) and 1.88° (refocusing). (B) SPGR (MM) using flip angle 0.06°. (C) bSSFP (MM) with flip angle 0.36°. (D) TSE (NM) with flip angle 26° (excitation) and 57° refocusing. (E) SPGR (NM) with flip angle 26°. (F) bSSFP (NM) with flip angle 26°. Shading artifacts close to the guidewire on (D) and (F) likely come from receiver effects. bSSFP, balanced steady‐state free precession; MM, maximum current modes; NM, null current mode; SPGR, spoiled gradient echo

Figure 6A though C shows coronal view projection images, normalized to maximum value, which were acquired using the visualization sequences. It can be seen that TSE provides the cleanest delineation of the guidewire with very little background contamination. In contrast, the bSSFP shows the guidewire but with contamination from banding artifacts that are bright for low flip angles rather than the more familiar dark bands usually seen when using higher flip angles. The SPGR also shows the guidewire; however, the background signal is greater than that of the TSE by 152%. The SNR ratio for each guidewire visualization technique was 114, 106, and 58 for the TSE, SPGR, and SSFP, respectively. The TSE demonstrates a better image quality and guidewire to background contrast.

**Figure 7:**
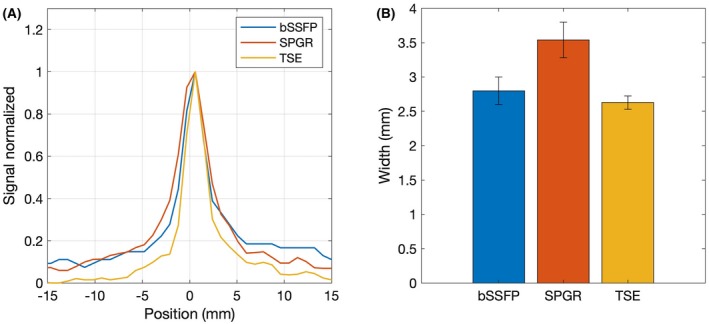
The guidewire width measured as the FWHM of a line profile through the guidewire in an axial image. (A) A curve is shown for TSE, SPGR, and bSSFP while imaging a straight guidewire and (B) the respective mean FWHM of 100 positions along the guidewire shaft, with error bars indicating plus or minus the SD

**Figure 8:**
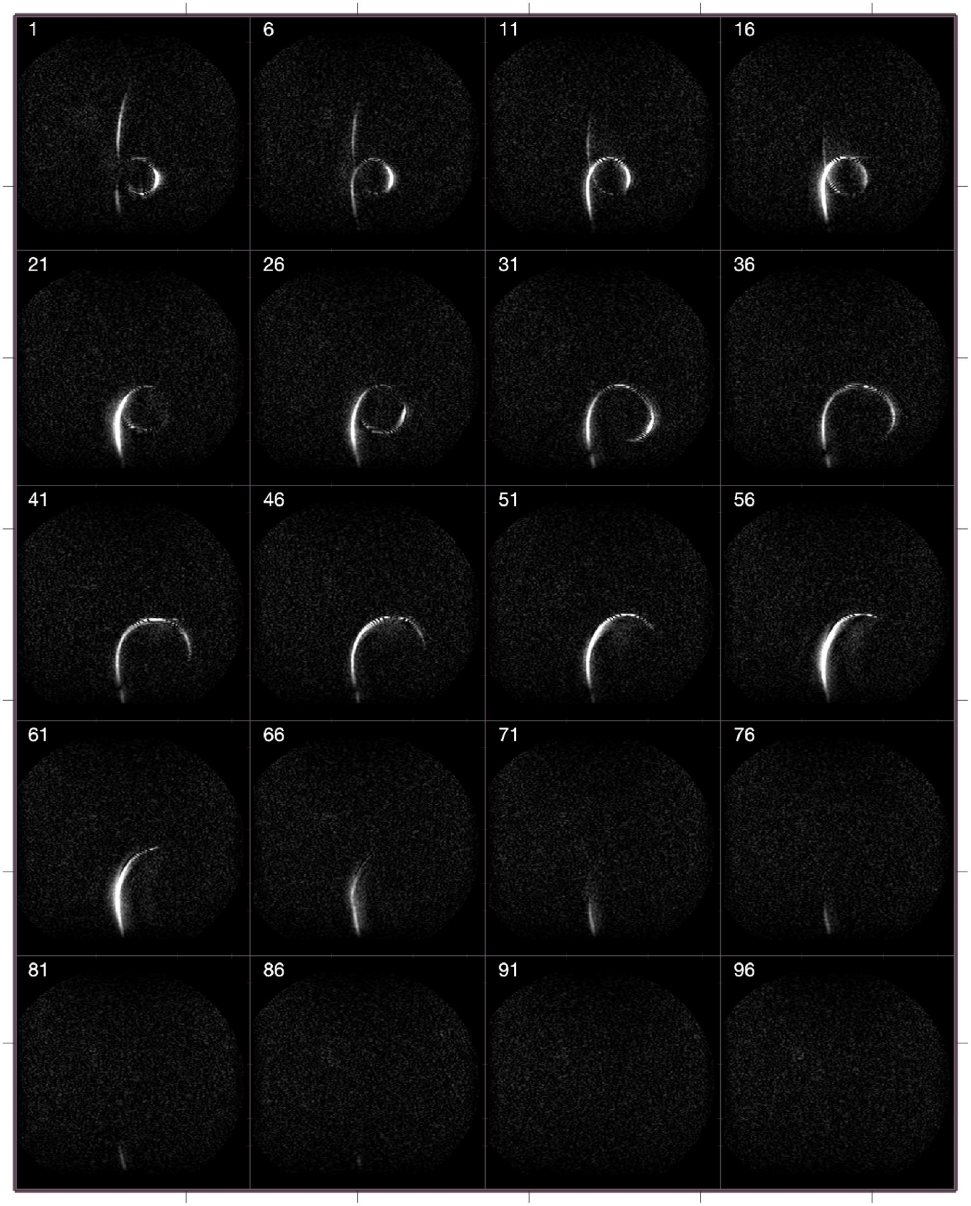
Frames from the real‐time acquisition at 2.5 fps while the guidewire is manually pulled out of the gel phantom. The frame number is shown in the upper left corner of every patch. Only the guidewire is visible, and the background signal is dark. Note that only a single static RF shim is used

Corresponding “tissue” visualization images generated using the shimmed NM excitations are shown in Figure 6, 7, 8, 9D through F. The guidewire is still visible in these images. This is hypothesized to be due to the guidewire having a similar (but uncontrolled) effect on the receiver sensitivity of the array coil and residual enhancement from currents outside the null point. Nevertheless, the background poly(acrylic) gel can clearly be visualized along with the circles array structure.

**Figure 9:**
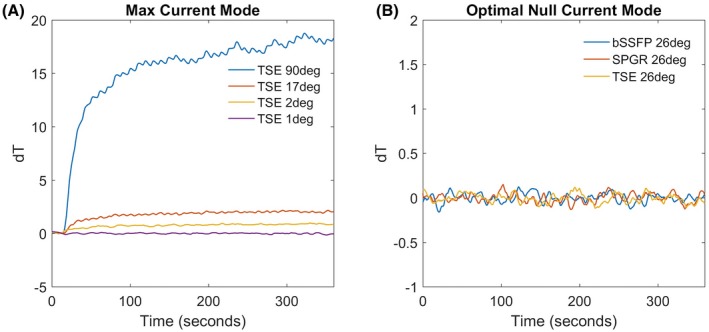
Temperature readings at the tip of the guidewire using optical temperature sensors during a 6‐min TSE scan with (A) MM and (B) optimal NM (the combination of all NM that result in a uniform B1 region). In (A), TSE with a nominal flip angle of 1° gives rise to a temperature elevation of only 0.02°C

In Figure 6F, banding artifacts are observed in the periphery of the image, which are well known to exist in balanced sequences and can be reduced with static field shimming.^41^ The *B*_0_ inhomogeneity around the guidewire was handled with localized second‐order shimming. In the null images, Figure 6D through F, the guidewire sometimes shows up as a signal void as in Figure 6E; and at other times, Figure 6D and F, the tip is present with signal and some of the shaft is void of signal. The guidewire visualization is not consistent across pulse sequences, leading to the conclusion that the receive enhancements seen in null images alone are not reliable enough to visualize the guidewire. Furthermore, these effects would be less appreciable in a heterogeneous background.

Figure 7 shows line profiles through the images from Figure 6A through C; the measured full width at half maximum (FWHM) for the guidewire is plotted. It is revealed that the TSE displays the narrowest guidewire width (Figure 7B). The FWHM mean of 100 points along the guidewire shaft is 2.8 mm (± 0.19), 3.5 mm (± 0.26), and 2.6 mm (± 0.09), respectively, for the bSSFP, SPGR, and TSE.

### 4.3 Real‐time visualization

Selected frames of the real‐time TSE acquisition of the guidewire being manually pulled out from the phantom are shown in Figure 8. The real‐time results are shown in Supporting Information Video S1. A frame rate of 2.5 fps was achieved using all available standard parameters in the pulse sequence definition. Although the coupling matrix can be changing during the guidewire pull, it was not updated throughout this experiment. However, with a single coupling measurement and RF shim setting, the guidewire was visible throughout its trajectory. Figure 3 depicts the guidewire geometry used while the guidewire was withdrawn from the phantom.

### 4.4 Heating tests

Temperature measurements made using a high SAR TSE sequence are shown in Figure 9. A temperature change of 18.0°C was measured for the MM at full amplitude (*θ* = 90°) and of 0.02°C with the MM at 1.1% (*θ* = 1°) amplitude used for guidewire visualization. The optimal combination of NMs used for anatomical imaging did not produce any detectable temperature increase when used with the TSE, SPGR, or bSSFP set to a nominal flip angle of 26 degrees. The $B_{1}^{+}$ field used in these tests was sufficiently high for anatomical visualization in the entire FOV. It was also noted that no temperature increase was detected at the point of hand contact while handling the guidewire from outside the phantom, which supports what is found in the literature.^20^

## 5 DISCUSSION

Parallel transmission offers a method for allowing guidewires to be “decoupled” or maximally coupled from the MRI transmission system. In this work, we propose to use such a system to effectively visualize these devices using the TSE pulse sequence configured in a way that maximally couples to them while transmitting at low power. Although this would be unsafe at normal power levels, our results show that the local *B*_1_^+^ fields are significantly enhanced by a factor of over 100 when maximum coupling mode is used, as shown in Figure 4. The use of standard pulse sequences with significantly reduced nominal flip angles therefore results in high signal from the blood adjacent to the guidewire and very little signal anywhere else. The fact that these sequences use very low amplitudes means the associated RF power is very small, and heating effects are insignificant. An added benefit of this approach is that the mode in which the transmit coil is used may be switched instantly (potentially on a pulse‐by‐pulse basis). Hence, “decoupled” mode excitations can be used for standard anatomical imaging with high RF power levels but no heating, and strongly coupled modes can be used for guidewire visualization at very low RF power levels, also with no heating, potentially within the same imaging sequence.

Simulations predicted that TSE sequences scaled to very low power levels would offer sharp visualization of wires in the maximum coupling mode, which was confirmed experimentally. Although bSSFP can offer higher SNR, the sequence suffers from severe “bright band” off‐resonance artifacts when used with very low flip angles, making these images less suitable for guidewire visualization. Heating tests showed that when run at very low power, MM excitations do not produce any measurable heating. Conversely, any required sequence can be used for visualization of background tissue in conjunction with a NM excitation, with no recorded heating at the guidewire tip, even when running at 100% (reported) SAR. Similar trends were observed in other experiments, including some using the same setup, others with a much larger phantom (~30 liters) and a local 8‐channel PTx surface coil (data not shown), and others using a much smaller phantom.^42^ The presented heating measurements were focused on the guidewire tip considering that in the literature this is the location where SAR and heating will manifest the highest.^20^, ^25^ Furthermore, when conducting test procedures, the interventionist manipulating the guidewire did not detect feeling any increases in temperature for any experiments conducted. This said, it was not possible to systematically monitor temperature along the guidewire’s length; therefore, we cannot rule out some unobserved heating, which is a limitation of the current study.

A limitation of using only NM excitation modes for background tissue visualization is that the efficiency of the RF coil is effectively reduced by removing the MM modes; thus, production of a homogeneous excitation within peak power limits is a challenge. In the present work, this limited achievable flip angles when using NM excitation; for example, for TSE the excitation was limited to 26° instead of 90°. Future work to address this will focus on the design of the RF transmitter coil because the generated field patterns are expected to influence this. Another issue in the use of metal wires is that receiver (i.e., $B_{1}^{-}$) coupling can also lead to image artifacts, as seen on Figure 6D, local to the guidewire. Image processing methods could potentially be used to reduce such effects because they are multiplicative to the image. Differential receiver‐related enhancements between receive channels and the presence of receiver‐related signal voids can be reduced by reconstructing array coil data using sum of squares, as detailed by Eryaman et al.^43^ The effects can be seen in comparing Figures 6D and F with Figure 6E, demonstrating both SENSE and sum‐of‐squares reconstructions. Hence, the NM imaging results in Figures 6D and F could likely be improved by an altered handling of receiver data in the reconstruction. Additionally, there may remain small currents on the full extent of the guidewire to produce transmit enhancement effects. This is because the null of the current is only guaranteed at the location of the current measurement. It is noted that these small remaining currents do not pose a heating risk, as seen in the results of the heating test in which the same protocol was used. A solution to this might be to place current sensors inside the dielectric medium to achieve a stronger null in the FOV.^44^

A potential issue with the proposed guidewire visualization approach is that the tip of the guidewire is less easily visualized than the shaft. Figure 8 and the supporting video file (Supporting Information Video S1) show that it is still possible to see the tip as it moves, but it is fainter than the rest of the guidewire. This is because the induced currents on the guidewire decay to 0 (or a very small value) at the tip. There is evidence that an optimal length of exposed guidewire tip of around 2 to 10 mm exists that increases guidewire tip visualization.^14^ In this work, we did strip insulation over the last 4 mm of the guidewire, although this was motivated by a desire to create a worst case heating risk. In practice, we would not advocate for purposely stripping insulating coatings because this would introduce a safety risk if currents are not correctly controlled, and exposure of a sharp metal tip may also risk puncture injuries. Others have investigated methods for visualization only of a catheter or guidewire tip, for example, by using a balloon‐tip catheter filled with gadolinium or air/CO_2_ 45, 46 or by using modified interventional devices with integrated tracking coils^47^; however, a drawback of these methods is that they can only visualize the tip (or the single position where the marker is placed). Hence, a hybrid solution using our proposed method along with a separate tip marker may prove most successful. Additionally, it has been noted elsewhere that visualizing the shaft alone and the close proximity to the tip may be sufficient for successful guidewire placement in vivo.^8^ Another implementation challenge is to keep the tip and shaft in plane and know when the tip or shaft has fallen outside the plane. A practical solution is to use a thick slice to encompass the guidewire. A computational alternative is to use multiple real‐time projections to reconstruct the three dimensional trajectory of the device in a fraction of a second and estimate the image slice containing the feature of interest.^48^

The number and positioning of current sensor(s) is critical both for practical reasons and safety. In this work, 2 sensors, both outside the phantom, were used to estimate currents on the inserted part of the guidewire; based on the temperature results, induced currents are nulled. However, it cannot be certain that the same conditions exist when the guidewire geometry changes. There may be instances when modes exist on the inserted length that are not fully sensed on the external length. It has been shown that a key variable is insertion length,^6^ which changes during an intervention, making it important to actively control and monitor induced currents both outside and potentially inside the dielectric (i.e., human body). Optimal sensor placement and RF coil design for this application are the subjects of current work.^44^ Alternative image‐based methods for measuring the coupling matrix have been proposed.^49^, ^50^

A feature of the proposed method is that the optimal nominal flip angle for visualization must be determined empirically because we expect guidewire visibility to be a function of the coupling to the coil, which is likely to vary. The inability to make adjustments continuously is a limitation of the current approach; however, in practice this may not prove to be a serious issue because the user could adjust this parameter while viewing the images in real time. Because guidewire visualization is achieved with very low RF power, the sweep through flip angles may be safely accomplished.

In future work, a faster frame rate will be investigated to visualize rapid guidewire movement with a goal of 7 fps. The methods used in this paper achieved adequate speed for a proof of concept using standard sequences. It is clear from Figures 6 and 8 that the guidewire‐only images are truly sparse; hence, there are many opportunities for acceleration using alternative sampling/reconstruction methods.

## 6 CONCLUSION

A method for visualizing a standard guidewire separate from the anatomy has been demonstrated by using a maximum coupling mode at low power, which is usually regarded as hazardous and omitted. TSE sequences at very low power were found to yield sharp delineation of the guidewire at reasonable SNR without severe off‐resonance artifacts present in balanced SSFP images.

## Supporting information


**VIDEO S1** The real‐time movie showing results of the TSE acquisition of the guidewire being manually pulled out of the phantom. A frame rate of 2.5 fps was achieved using all available standard parameters in the pulse sequence definition. As the guidewire is pulled out the coupling matrix can be changing, but this was not accounted for in the present experiment
